# Heterometal Grafted Metalla-ynes and Poly(metalla-ynes): A Review on Structure–Property Relationships and Applications

**DOI:** 10.3390/polym13213654

**Published:** 2021-10-23

**Authors:** Rayya A. Al-Balushi, Ashanul Haque, Idris J. Al-Busaidi, Houda Al-Sharji, Muhammad S. Khan

**Affiliations:** 1 Department of Basic Sciences, College of Applied and Health Sciences, A’Sharqiyah University, P.O. Box 42, Ibra 400, Oman; 2 Department of Chemistry, College of Science, University of Hail, Ha’il 81451, Saudi Arabia; 3 The Directorate General of Private Schools, Ministry of Education, P.O Box 3, Muscat 100, Oman; idris.albusaidi.oman@gmail.com; 4 Department of Chemistry, Sultan Qaboos University, P.O. Box 36, Al-Khod 123, Oman; houda.sultan91@gmail.com

**Keywords:** metalla-ynes, poly(metalla-ynes), polymers, opto-electronic, photo-physical

## Abstract

Metalla-ynes and poly(metalla-ynes) have emerged as unique molecular scaffolds with fascinating structural features and intriguing photo-luminescence (PL) properties. Their *rigid-rod* conducting backbone with tunable photo-physical properties has generated immense research interests for the design and development of application-oriented functional materials. Introducing a second *d*- or *f*-block metal fragment in the main-chain or side-chain of a metalla-yne and poly(metalla-yne) was found to further modulate the underlying features/properties. This review focuses on the photo-physical properties and opto-electronic (O-E) applications of heterometal grafted metalla-ynes and poly(metalla-ynes).

## 1. Introduction

Metalla-ynes and poly(metalla-ynes) are *rigid-rod* type molecular systems in which one or more transition metal ions are connected to the alkynyl unit via σ-linkage [1,2]. Owing to their excellent photo-luminescence (PL) properties, structural features and diverse applications, this class of materials has received significant attention in the last two decades. In this class of materials, the electronic structure and properties are a function of the overall chemical composition of the material, i.e., type of organic spacers, no. of alkynyl units, metal fragments, and auxiliary ligands attached to the metals [1,3,4,5]. For instance, the introduction of a heavy transition metal ion into the backbone of organic poly-yne core induces spin–orbit coupling (SOC) and facilitates intersystem-crossing (ISC) leading to efficient photo-luminescence quantum yield (PLQY) [6,7,8,9]. Similarly, when an electron-rich (donor, D) and an electron-deficient (acceptor, A) organic spacers are introduced, it creates a strong intramolecular D-A interaction leading to absorption and emission extending from the visible to near-infrared (NIR) region of the spectrum [10,11,12,13,14]. The impact of topology (point of attachment) on the photo-physical properties (such as isomerization) was also established [15,16]. Several functional materials have been developed with potential application in molecular electronics, photo-voltaic, organic light-emitting diodes (OLEDs), bioimaging, catalysis, etc. [3,17,18]. 

**Figure 1:**
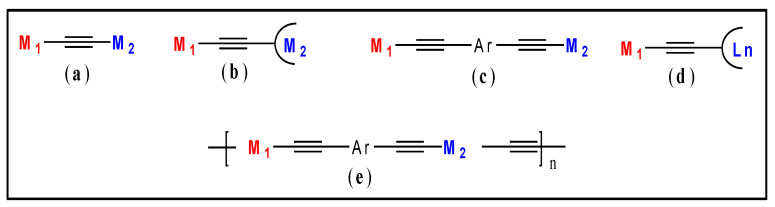
General chemical structure of some of the *d-d* and *d*-*f* fragments containing heterometallic complexes. M_1_ and M_2_ = transition metal ions (**a**), Ln = a lanthanide core (**b**), Ar = conjugated carbocyclic/heterocyclic spacers and (**c**) semi-circle = bi- (**d**) or polydentate donor ligands (**e**).

Among the various strategies adopted by researchers, the introduction of a second metal ion into the main-chain or as side-chain of the metalla-ynes and poly(metalla-ynes) is a judicious way to fine-tune their PL features. Several complexes in which *d*-block and *f*-block metal fragments are connected through conjugated [19] and non-conjugated [20] linkers were reported in the literature. It is to be noted that the high conductivity, preservation of conjugation through the metal fragment, high triplet yield, etc. make “*d*” fragment a matter of choice for the metalla-ynes and poly(metalla-ynes) [21]. The heterometallic complexes bearing conjugated metalla-yne fragments often display modulated properties due to the synergistic effect of the two different metal ions [2,3,22,23,24,25]. For example, it is reported that combining two transition metal (*d*-*d*) fragments in an alkynyl complex affects the energy of the frontier molecular orbitals, emission lifetime [26], and, to some extent, solid-state packing [27]. Similarly, it was demonstrated that in mixed metal alkynyl complexes, the metalla-yne core sensitizes NIR lanthanide luminescence [28] and improves decay lifetime (from µs to ms) [29] via *d* → *f* energy transfer pathways. Inspired by this heterometallic cooperativity, numerous complexes were reported with *d*-*d* and *d*-*f* combinations [20,29,30,31,32,33,34,35,36,37]. We present herein a review of the photo-physical properties and O-E applications of heterometal grafted metalla-ynes and poly(metalla-ynes). In this paper, we considered only those examples in which *d*-*d* and *d*-*f* fragments are separated by one or more alkynyl units (Figure 1). Both, the main-chain, and side-chain metalla-ynes and poly(metalla-ynes) were considered to get a clear picture.

## 2. Impact on Properties

### 2.1. Main-Chain Systems

Transition metal complexes display a multitude of colorful optical (absorption/emission) and interesting magnetic properties due to the presence of metal ions in different oxidation states [38]. Moreover, multi-dimensional applications of transition metal complexes are well established [39]. It is well demonstrated that when one or more transition metal ions (decorated with suitable auxiliary ligands) are embedded in an organic framework via σ-linkage, new conducting materials are realized with limitless features and properties. Such materials, commonly known as metalla-ynes or poly(metalla-ynes), were studied and reviewed by several groups [40]. Both homo- and heterometallic σ-acetylide metal complexes with one or more types of transition metal ions are known in the literature, with a majority on homometallic systems [1,2]. The combination of two transition metals provides an effective way to realize new materials with unique structural features and improved properties such as high solubility and transparency [41]. Although heterometallic poly-ynes containing Ni/Pt and Pd/Pt have been known for a long time [42,43], focus on using other metals has been sparse, possibly due to the challenging synthesis of suitable monomers and/or polymers. In this sub-section, we exemplify the structure and properties of heterometallic molecular systems while clusters [44] are beyond the scope of this paper. Attempts were made to include recent references that have not been covered before [25].

**Figure 2:**
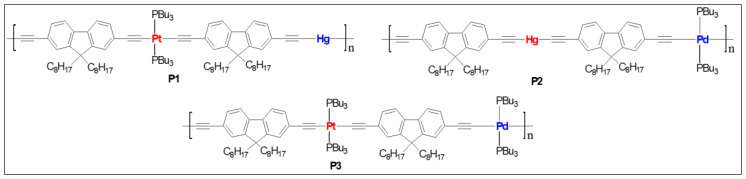
Heterometallic poly(metalla-ynes) **P1**–**P3**.

Dixneuf and co-workers [45] reported the first one dimensional Ru(II)/Pd(II) mixed metalla-yne as a yellow oligomer in good yield (84%) and moderate chain length (*M*_w_ = 14,800, *M*_n_ = 7800). Basic structural characterization showed the formation of mixed metalla-yne but no photo-physical properties were reported. Wong and co-workers [46] reported the very first example of *soluble* rigid-rod heteronuclear Pt(II)/Hg(II) poly(metalla-yne) (**P1**, Figure 2) and their corresponding model complexes. The reported white polymer exhibits excellent thermal stability (*T*_d_ = 366 ± 5 °C), and polydispersity (*M*_w_ = 29,790, *M*_n_ = 15,704, Table 1). The fluorescence quantum yield of the heterobimetallic polymer (Φ_f_ = 0.52 in DCM, Table 1) was found to be lower than the homometallic *trans* polymers (Φ_f_ = 2.53 for Hg(II) and 0.62 for Pt(II) in DCM) but higher than the *cis* counterpart (Φ_f_ = 0.1 in DCM). Similarly, the heterometallic Pt(II)/Hg(II) complex showed a weak triplet emission at room temperature (RT) and a slightly stronger optical power limiting (OPL) performance than the homo-nuclear Hg(II) complexes. The transparency window of the poly-ynes in the visible regime coupled with their OPL performance was achieved by interrupting π- conjugation via copolymerization with Hg and tuning the Pt geometry (*cis* and *trans*).

**Figure 3:**
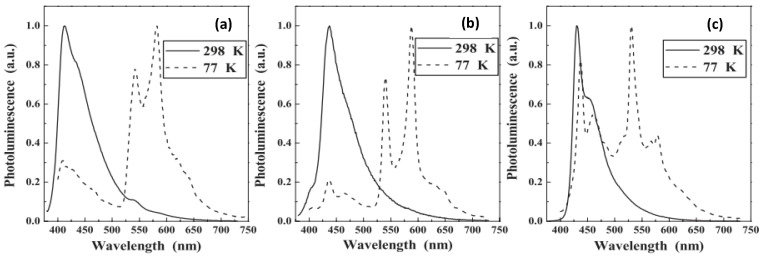
Photoluminescence (PL) spectra at 298 K and 77 K in CH_2_Cl_2_ for (**a**) **P1**, (**b**) **P3**, and (**c**) **P2**. Reproduced with permission from ref. [47].

A comparative study on homo- and heterometallic poly-ynes (**P1**–**P3**, Figure 2) indicated that Hg(II) and Pt(II) containing poly-ynes are better candidates for OPL than the corresponding Pd(II) poly-ynes as the former display absorption maxima below 400 nm [47]. It was shown that the heterometallic complex **P1** shows better transparency (maximum absorption wavelength (*λ_max_*) = 386 nm, Table 1) than that of homometallic Pt(II) complexes (Figure 3). Theoretical calculations suggested that in heterometallic complexes, the contribution of *d_π_* orbitals to the highest occupied molecular orbital (HOMO) was more from Pd/Pt than the Hg. Conversely, the contribution of *p_π_* orbitals to the lowest unoccupied molecular orbital (LUMO) was more from the Hg fragment, in line with the earlier studies [46].

**Figure 4:**
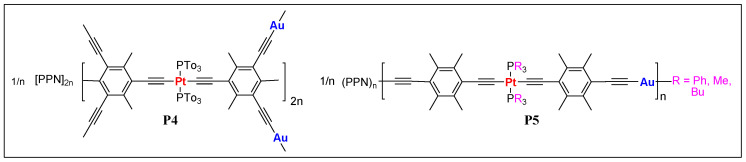
Pt(II)/Au(I) heterometallic anionic poly(metalla-ynes) **P4** and **P5**. (PPN = bis(triphenylphosphine)iminium cation).

In 2005, Vicente et al. [48] reported the very first examples of Pt(II)/Au(I) heterometallic anionic poly(metalla-ynes) **P4** and **P5** (Figure 4). The polymers, obtained by reacting Pt(II) *bis*- or *tetra* acetylides with PPN[Au(acac)_2_] (PPN = *bis*(triphenylphosphine)iminium cation, acac = acetylacetone) were poorly characterized due to the extremely low solubility. The only polymer **P5** (R = Bu) was soluble and could be characterized by nuclear magnetic resonance (^1^H, ^13^C, and ^31^P NMR) and gel permission chromatography (GPC) techniques, which showed comparatively good chain length of the polymers (up to 667 units).

**Figure 5:**
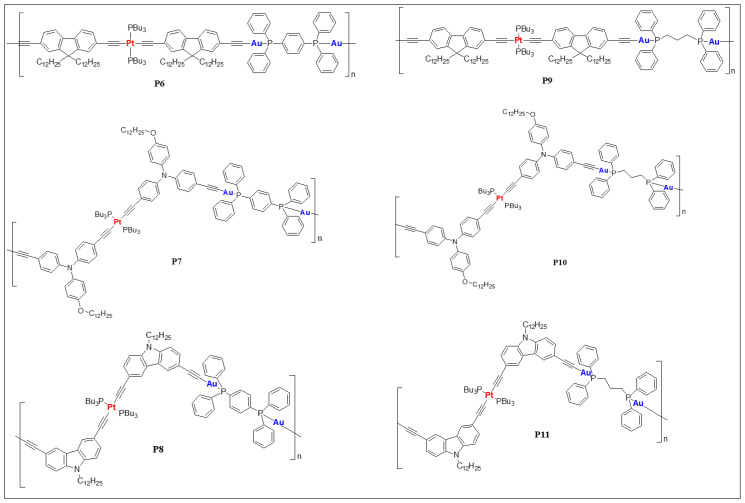
Heterometallic Au(I)–Pt(II) poly(metalla-ynes) (**P6**–**P11**) reported by Wong et al. [22].

Wong and co-workers [22] reported heterometallic Au(I)–Pt(II) poly(metalla-ynes) (**P6**–**P11**, Figure 5), in which the impact of merging two different metals can be clearly observed (Table 1). Compared to the homometallic systems, Au(I)–Pt(II) poly(metalla-ynes) showed blue-shifted absorption maxima and cut-off absorption wavelengths in solution slightly shifted with respect to monomeric Pt(II) complexes. Merging two different metallic cores also significantly improved the transparency of the resulting material, which was attributed to the weak metal-ligand interactions and conjugation interruption by auxiliary ligands.

### 2.2. Side-Chain Systems

#### 2.2.1. *d-d* Metal-Containing Systems

**Figure 6:**
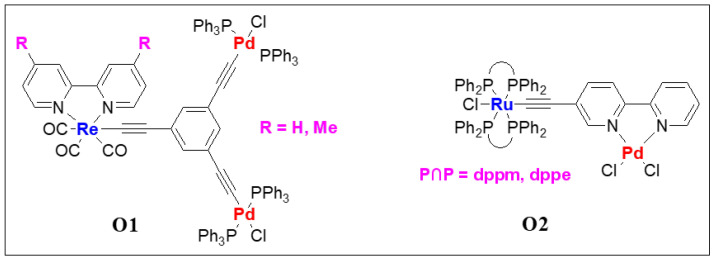
Re(I)/Pd(II) hetero-trimetallic (**O1**) and Ru(II)/Pd(II) heterometallic (**O2**) complexes.

Compared to the main chain heterometallic systems, dimers and polymers with second organometallic fragments attached via chelating ligand are more common in the literature. Several oligo- and poly(metalla-ynes) bearing heterometallic fragments were synthesized and studied in the past. In this section, we discuss some pertinent examples of both small and large molecular systems with two or more types of metal ions. As observed in the earlier examples, the inclusion of a second metal affects the PL properties. Among *d*-*d* combinations, the use of Ir(III) and Re(I) with Pt(II) metalla-ynes is very common as the mixed-metal systems show improved photo-physical behavior [49]. It is well established that the photo-physical properties of a material are the function of the molecular structure of the main-chain and pendant ligands. Studies on heterometallic branched complexes indicated that the absorption and emission energies shift to the red upon the incorporation of the Pd(II) fragment moiety [50]. Compared to monometallic Re(I) complexes (maximum absorption wavelength ($\lambda_{max}^{abs}$) = 408–418 nm), trimetallic complexes **O1** (R = H or Me, Figure 6) exhibit low energy transition at $\lambda_{max}^{abs}$ = 416–426 nm in solution (Table 2). Similarly, in tetrahydrofuran (THF) solution at RT, Re(I) complexes showed maximum emission wavelength ($\lambda_{max}^{em}$) at 625–636 nm (lifetime, τ_0_ = < 0.1 μs), while Pd(II)/Re(I) complexes emitted at $\lambda_{max}^{em}$ = 628–639 nm (τ_0_ = < 0.1 μs, Table 2). Heterometallic complex **O2** (Figure 6*)* bearing Pd(II)/Ru(II) core exhibits highly red shifted absorption band (Table 2) compared to monometallic Ru(II)-counterpart [51].

**Figure 7:**
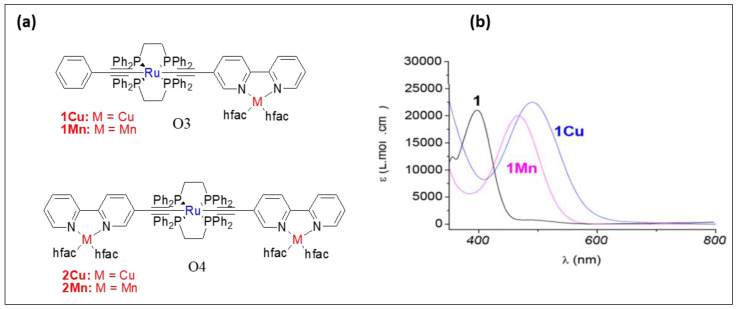
(**a**) Structures of bimetallic and trimetallic Ru/Cu and Ru/Mn complexes **O3** and **O4** and (**b**) the optical absorption spectra (in CH_2_Cl_2_) of **O3** when M = Cu (1Cu) and Mn (1Mn) along with the corresponding monometallic Ru-bipyridyl compound (**1**). Reprinted (adapted) with permission from Di Piazza, E.; Boilleau, C.; Vacher, A.; Merahi, K.; Norel, L.; Costuas, K.; Roisnel, T.; Choua, S.; Turek, P.; Rigaut, S., Ruthenium carbon-rich group as a redox-switchable metal coupling unit in linear trinuclear complexes. Inorg. Chem. 2017, 56, (23), 14540–14555. Copyright 2017 American Chemical Society.

Piazza and coworkers [52] assessed the photo-physical and magnetic properties of Ru(II)/Cu(I) and Ru(II)/Mn(I) couples **O3** and **O4** (Figure 7). Interestingly, a long-distance magnetic coupling between the terminal Cu(II) units through Ru(II) fragment was noted. Moreover, heterometallic systems displayed low energy bands in the visible region (Table 2). Cu(II) complexes exhibit higher thermal stability compared to Mn(I) complexes.

**Figure 8:**
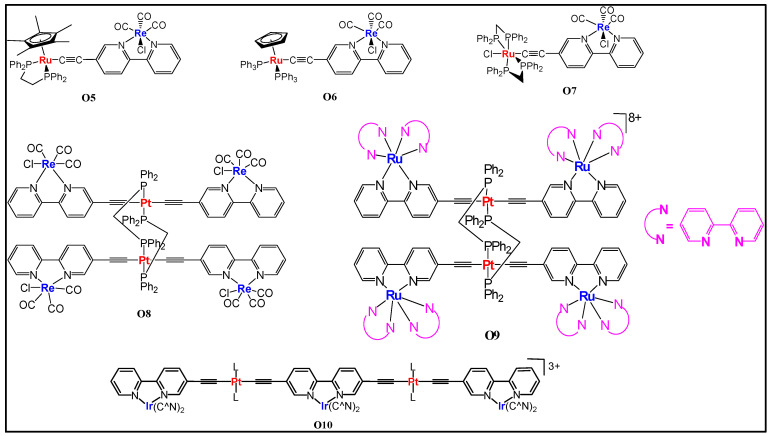
Structures of the *d*-*d* and *d*-*f* type heterometallic complexes **O5−O10**.

In contrary to this, theoretical and experimental studies suggest that the two metal centers in binuclear heterometallic Ru(I)/Re(I) complexes **O5**–**O7** (Figure 8) are weakly coupled [27]. Chen et al. [26] found that the insertion of one or more heterometal (Re/Ru) reduces the π* energy level in the ethynyl bipyridyl ligand in platinaynes and thus alters the photo-physical properties. For example, complexes (**O8** and **O9**, Figure 8) showed a red-shift in optical absorption and longer lifetime (in µs) compared to monometallic platinaynes (Table 2). Complex with Pt/Ru couple ($\lambda_{max}^{abs}$ = 504 nm, $\lambda_{max}^{em}$ = 658 nm and τ_0_ = < 0.1 μs) showed red shifted absorption and emission compared to Pt/Re $\lambda_{max}^{ab}=\;427\;\mathrm{nm},\;\lambda_{max}^{em}$ = 595 nm and τ_0_ = < 1.5, 0.22 μs) and Pt ($\lambda_{max}^{ab}=\;392\;\mathrm{nm},\;\lambda_{max}^{em}$ = 540 nm and τ_0_ = < 0.1 μs) complexes.

In addition to these small molecular systems, several polymeric complexes bearing *d*-*d* metal fragments were also investigated [53]. Complex **O10** (Figure 8) is an example of a highly emissive (Table 2) pentanuclear complex containing Pt(II) and Ir(III) fragments [54]. An efficient triplet energy transfer between the terminal and central Ir(III) cores through the Pt(II) moiety was reported in such systems.

**Table 2:** Photoluminescence (PL) data of some selected heterometallic metalla-ynes **O1−O4** and **O8−O10**.

Code	Metals	*λ_abs_*(nm)	*λ_ems_*(nm)	Lifetime of S_1_(τ^a^, µs)	Lifetime of T_1_(τ^b^, µs)	Φ (%)	Ref.
**O1** (R = Me)	Pd/Re	236, 284, 336sh, 416	628 ^a^, 589 ^b^	˂0.1	0.73	-	[50]
**O1** (R = H)	Pd/Re	238, 284, 334sh, 426	639 ^a^, 594 ^b^	˂0.1	0.62	-	[50]
**O2** (dppm)	Pd/Ru	386	-	-	-	-	[51]
**O2** (dppe)	Pd/Ru	389	-	-	-	-	[51]
**O3**	Ru/Cu	308, 494	-	-	-	-	[52]
**O3**	Ru/Mn	308, 468	-	-	-	-	[52]
**O4**	Ru/Cu	308, 494	-	-	-	-	[52]
**O4**	Ru/Mn	306, 468	-	-	-	-	[52]
**O8**	Pt/Re	271, 382, 427	595	1.5, 0.22	-	0.0018	[26]
**O9**	Pt/Ru	243, 291, 360, 504	658	<0.1	-	0.045	[26]
**O10**	Pt/Ir	255, 315, 415, 435	560 ^a^, 613 ^a^, 663 ^a^,550 ^b^, 595 ^b^, 651 ^b^	2.3	2.5, 1.9	3.3	[54]

*λ_abs_*: absorbtion wavlength peaks, *λ_ems_*: emission wavelength peaks, sh: shoulder, a: measured at 298 K, b: measured at 77 K, Φ: quantum yield.

**Figure 9:**
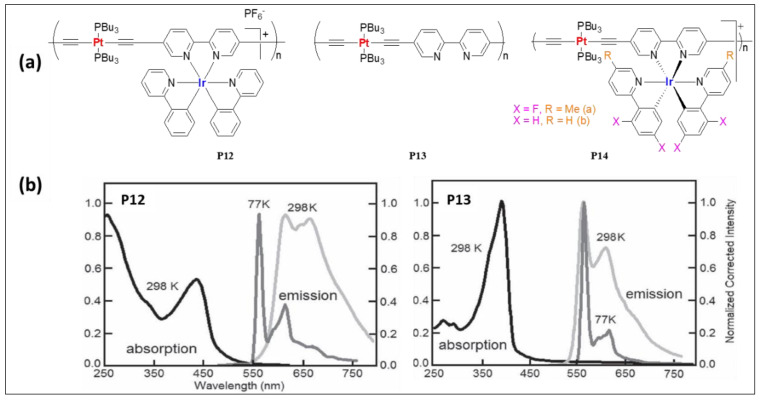
(**a**) Mono- and bi-metallic Pt(II)/Ir(III) polymers (**P12**–**P14**). (**b**) Absorption (298 K) and emission spectra of **P12** and **P13** at 298 and 77 K. Reproduced with permission from ref. [55].

Harvey and coworkers [55] prepared a series of mono- and bimetallic Pt(II)/Ir(III) complexes (**P12**–**P14**, Figure 9a) and assessed their photo-physical properties. The photophysical features of the heterometallic complexes were found to be a hybrid of the monometallic complexes used. For instance, Ir(III)-containing 5,5′-bisacetylide complex (Φ = 1.6%, τ = 0.09 μs) showed structureless emission maxima at 638 nm while Pt(II) dimer (Φ = 13.7%, τ = 39.2 μs) showed a blue-shifted structured emission at 561 nm. The introduction of a second luminescent fragment in Pt(II) complex (Φ = 13.7%, τ = 39.2 μs) led to Pt/Ir (Φ = 4%, τ = 1.33 μs) dimer with red-shifted emission at 623 nm. Under similar conditions, polymer **P13**, which is a Bipy containing Pt(II) poly-yne exhibits emission at 561 nm (Φ = 12.8%, τ = 9.2 μs, Table 3). Ir-containing polymer **P12** showed emission at 617 nm (Φ = 2.6%, τ = 1.22 μs, Table 3) (Figure 9b). Later the same group [56] compared the photo-physical and electrochemical properties of complex **P14** (Figure 9a). Upon fluorination of the pendant ligand, the nature of the excited state remains the same; however, there were some changes in the absorption and in emission profiles (Table 3).

**Figure 10:**
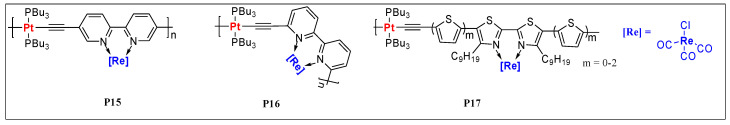
Re(I)-coordinated Pt(II) poly(metalla-ynes).

In the past, we demonstrated that the topology of alkynylated π-conjugated ligands plays an important role in determining electron transfer and other processes [57]. For example, it was found that the Pt(II) acetylide attached to different positions of azobenzene [15] and stilbene [16] exhibit different levels of isomerization. Later, we found that the inclusion of Re(I) core in Pt(II) di-ynes and poly-ynes alters the optical properties of the complexes, the extent of which depends on the topology of the bipyridine (Bipy) ligand (i.e., 5,5′- or 6,6′ systems, **P15**–**P16**, Figure 10 and Table 3) [33]. Like other reports, we also noted that the 5,5′-bisalkynyl systems are much better than the 6,6′- counterparts. Wong and co-workers [58] also reported that the coordination of a Re(I) unit, as well as the number of thienyl rings, plays a central role in determining the PL properties of poly(platina-yne) **P17** (Figure 10, Table 3).

**Table 3:** Photoluminescence (PL) data of selected heterometallic poly(metalla-ynes) **P12–P19**.

Code	Metals	Molecular Weight (× 10^4^)		*λ_abs_* (nm)	*λ_ems_* (nm)	Lifetime of S_1_ (τ^a^, ns)	Lifetime of T_1,_ (τ^b^, µs)	Φ (%)	E_g_ (eV)	Ref.
		*M*_w_	*M*_n_							
**P12**	Pt/Ir	1.33	1.18	250, 280, 340, 435	617 ^a^, 563 ^b^	1220	5.67	2.60	-	[55]
**P13**	Pt	1.22	2.48	253, 270, 295, 390	561 ^a^, 562 ^b^	9200	70	12.80	-	[55]
**P14**a	Pt/Ir	-	-	250, 260, 315, 415, 485	628 ^a^, 547 ^b^, 592 ^b^, 640 ^b^	800	4.81	1.0	-	[56]
**P14**b	Pt/Ir	-	-	250, 280, 340, 435	555 ^a^, 617 ^a^, 655 ^a^, 558 ^b^, 596 ^b^, 654 ^b^	2500, 1900	5.7, 3.3	2.6	-	[56]
**P15**	Pt/Re	6.1	7.7	343, 419, 448	-	-	-	-	-	[33]
**P16**	Pt/Re	5.5	8.3	276, 300, 324, 388, 402	-	-	-	-	-	[33]
**P17** (m = 0)	Pt/Re	1.3	3.8	230, 306, 539	582 ^a^	0.22	-	0.05	2.18	[58]
**P17** (m = 1)	Pt/Re	1.0	1.9	229, 362, 548	659 ^a^	0.44	-	0.19	1.95	[58]
**P17** (m = 2)	Pt/Re	0.9	1.9	229, 400, 552	729 ^a^	0.34	-	0.10	1.85	[58]
**P18**	Pt/Ir	1.3	-	229, 260, 287, 379, 400, 446, 465, 487	549 ^a^, 588 ^a^, 552 ^b^, 599 ^b^	110	10.82	12.3	2.44	[49]
**P19**	Pt/Ir	1.1 × 10^4^	-	229, 251, 283, 383, 400, 474, 502	577 ^a^, 625 ^a^ 577 ^b^, 631 ^b^	510	12.12	4.80	2.39	[49]

*M_w_*: average weight molecular weight, *M_n_*: number weight molecular weight, *λ_abs_*: absorption wavelength peaks, *λ_ems_*: emission wavelength peaks, sh: shoulder, a: measured at 298 K, b: measured at 77 K, Φ: quantum yield, E_g_: energy gap.

#### 2.2.2. *d-f* Metal-Containing Systems

**Figure 11:**
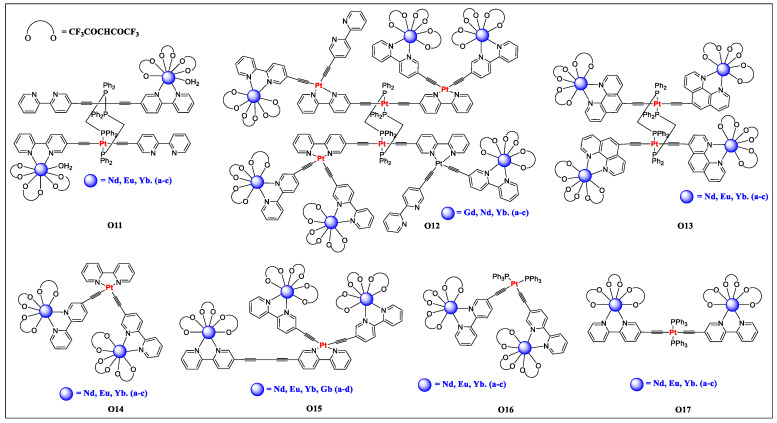
Acetylide-functionalized diimine-based hetero-multimetallic complexes with different lanthanides metal ions (**O11**–**O17**).

In contrast to *d*-block elements, *f*-block lanthanides (Ln) display fascinating optical (colorful sharp line-like emission spectra with large Stokes shifts and long lifetime) and magnetic properties [32,59,60,61]. Owing to these features, they serve as an excellent dopant for the fabrication of display devices and probes for cellular imaging. However, it is often overshadowed by the low molar absorption coefficients (ε ~ 0.1–10 M^−1^·cm^−1^) of Ln(III) complexes as *f-f* electronic transitions are forbidden by parity and spin selection rules. To overcome this limitation, coordination of transition metal organometallic complexes was proposed as it would facilitate efficient electronic energy transfer (EET) via the *antenna effect* [62,63,64,65,66]. In addition to the synergistic effect of these different blocks of metals, judicious selection of coordinating ligand, the excitation wavelength could be extended to the visible region [64]. In this context, π-conjugated arylethynyl spacer covalently linked to transition metal is an excellent choice [67,68,69]. Exploiting this concept, several hetero-multimetallic Ln(III) complexes incorporating Pt(II) acetylide chromophore were reported [25,32,34]. It was noted that Pt(II) acetylide complex strongly sensitizes the Ln(III) in the Vis-NIR region with a high Φ_f_ value [66,70]. In complexes (**O11**–**O17**, Figure 11, Table 4), an efficient energy transfer to Ln(III) ions takes place and the separation between Pt—Ln played an important role [28,71,72,73]. Regardless of Pt(II) isomer configuration (*cis* or *trans*) the low energy phosphorescence from dπ_(Pt)_→π*_(C≡C-Bipy/phen)_ ^3^MLCT excited states were quenched indicating efficient energy transfer from the Pt(II) acetylide chromophore to lanthanide centers.

**Figure 12:**
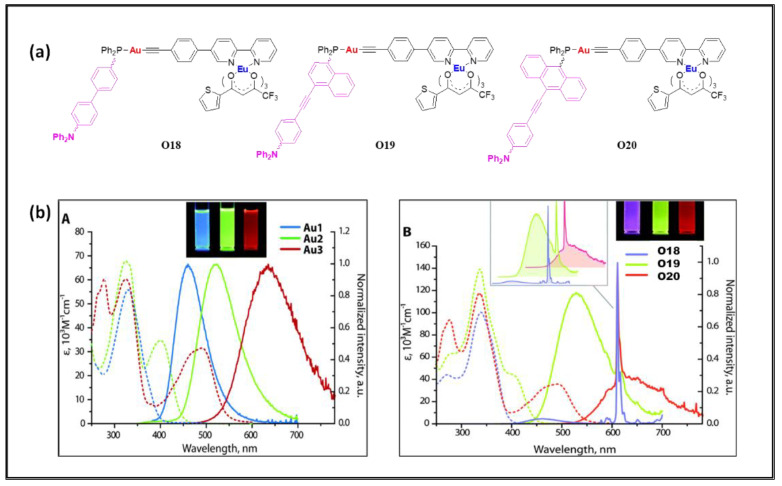
(**a**) Bipyridine-based Au(I) complexes incorporating Eu(III) β-diketonate fragment. (**b**) UV-vis absorption (dashed lines) and normalized emission (solid lines) spectra of metallo-ligands Au1–Au3 (A), and dyads **O18**–**O20** (B) the photographs show the corresponding solutions under UV light. Reproduced from ref. [74] licensed under a Creative Commons Attribution Non-Commercial 3.0 Unported License.

Belayev and coworkers [74] reported Au(I)/Eu(III)-based dually luminescent D–π–A type complexes **O18**–**20** (Figure 12a) with solvatochromic features (Figure 12b). In these heterometallic complexes, different π-extended phosphines ligands attached to Au(I) center were tested. It was noted that the partial energy transfer from phosphine → Eu(III) and efficient energy transfer from β-diketonate → Eu(III) were the main causes of dual emission (Table 4).

**Figure 13:**
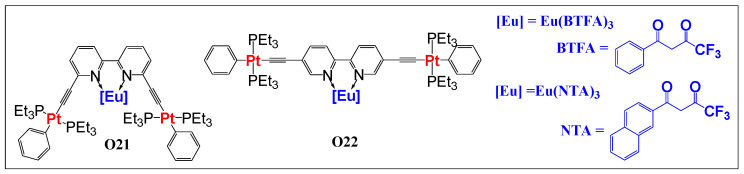
“*d*-*f*-*d*” type Eu(III)-co-ordinated Pt(II) di-ynes.

We recently investigated the structural and optical properties of Bipy-based Pt(II)/Eu(III) heterometallic complexes **O21** and **O22** (Figure 13, Table 4) [32,34]. In line with the other studies, we also found an efficient, but topology-dependent, *d → f* energy transfer in the complexes. Whereas complex **O21** exhibited typical Eu(III)-based red emission, complex **O22** displayed dual emission (red and green) with excitation in both the UV and Vis regions. Moreover, complexes exhibited longer lifetime (τ_obs_) than similar complexes reported in the literature [28,75].

**Table 4:** Photoluminescence data of heterometallic metalla-ynes **O11−O22** and **O24−O29**.

Code	Metals	*λ_abs_* (nm)	*λ_ems_* (nm)	Lifetime of S_1_ (τ^a^, µs)	Φ (%)	Ref.
**O11**a	Pt/Nd	-	1060^a^	-	0.84	[28]
**O11**b	Pt/Eu	-	615 ^a^	250.60	24	[28]
**O11**c	Pt/Yb	-	980	12.20	5.65	[28]
**O12**a	Pt/Gd	-	563	0.84	0.07	[71]
**O12**b	Pt/Nd	-	1060	weak	-	[71]
**O12**c	Pt/Yb	-	980	12.10	0.061	[71]
**O13**a	Pt/Nd	-	1060	-	0.2	[28]
**O13**b	Pt/Eu	-	615	33.60	0.5	[28]
**O13**c	Pt/Yb	-	980	12.90	0.635	[28]
**O14**a	Pt/Nd	304, 320, 363, 401	1061	-	-	[72]
**O14**b	Pt/Eu	301, 323, 365, 400	613	105	5.4	[72]
**O14**c	Pt/Yb	295, 322, 365, 400	980	11.8	6.3	[72]
**O15**a	Pt/Nd	304, 329, 358, 381, 430	1060	-	-	[72]
**O15**b	Pt/Eu	299, 329, 357, 382, 430	613	164	1.7	[72]
**O15**c	Pt/Yb	294, 328, 357, 382, 430	980	10.9	0.54	[72]
**O15**d	Pt/Gd	303, 329, 358, 383, 430	572	0.40	2.4	[72]
**O16**a	Pt/Nd	228, 297, 371	423, 1061	<0.01	-	[73]
**O16**b	Pt/Eu	228, 303, 375	420, 613	<0.01, 109.5	1.4	[73]
**O16**c	Pt/Yb	228, 297, 371	428, 980	<0.01, 12.6	0.63	[73]
**O17**a	Pt/Nd	229, 305, 372	421, 1060	<0.01	-	[73]
**O17**b	Pt/Eu	230, 303, 375	415, 613	<0.01, 216.3	1.0	[73]
**O17**c	Pt/Yb	228, 293, 384	448, 980	<0.01, 12.7	0.64	[73]
**O18**	Au/Eu	271, 339	460, 580, 592, 611, 654, 700	0.0022, 280.00	15	[74]
**O19**	Au/Eu	280, 337, 404	525, 611	0.0017, 434.00	21	[74]
**O20**	Au/Eu	276, 336, 495	611, 635, 705	0.0041, 404.10	16	[74]
**O21**	Pt/Eu	325	550–725	590	54	[32]
**O21**	Pt/Eu	325	550–725	410	29	[32]
**O22**	Pt/Eu	416	425–531, 550–725	39.52, 519.86	39	[34]
**O24**	Ir/Eu	242, 283, 343	578, 590, 615, 684, 697	0.780, 0.116, 0.51, 0.095	-	[76]
**O24**	Ir/Gd	242, 285, 338	560	1.1, 0.45 0.64, 0.22	4.8	[76]
**O25**	Ir/Eu	241, 292, 357	616	1.24, 0.168	-	[76]
**O25**	Ir/Gd	242, 292, 358	595	1.26, 0.233	2.6	[76]
**O26**	Au/Re	243, 265, 305, 366	592	-	-	[77]
**O27**	Au/Re	240, 278, 3.19, 357	576	-	-	[77]
**O28**	Au/Re	237, 271, 318, 352	607	-	-	[77]
**O29**	Au/Re	368	614	1060	30.4	[69]

*λ_abs_*: absorption wavelength peaks, *λ_ems_*: emission wavelength peaks, sh: shoulder, τ^a^: lifetime measured at 298 K, Φ: quantum yield.

## 3. Applications

**Figure 14:**
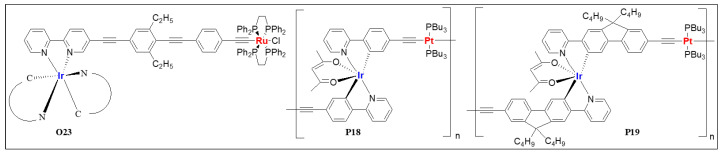
Heterometallic poly-ynes (**O23**, **P18** and **P19**) for NLO and OLED applications.

It is clear from the above discussion that the optical properties of homo- and heterometallic metalla-ynes and poly(metalla-ynes) are highly sensitive to the type of metal ions, number of π-linkages, spacers and end-groups. Despite this knowledge, relatively less attention has been focused on application-oriented studies. From the application point of view, complex **O23** (Figure 14) is the first example of a heterometallic complex featuring an NLO response [78]. This complex was reported to be a two-photon absorber (2PA) as well as an excited-state absorber under two different conditions. Heterometallic polymers (**P1**–**P3**, Figure 2) were reported as promising OPL candidates [47]. Among the tested heterometallic poly(metalla-ynes), Hg(II)/Pt(II) exhibit best optical-limiting thresholds (0.07 J·cm^–2^ at 92% linear transmittance) followed by Pd/Pt (0.35 J·cm^–2^) and Pd/Hg (0.75 J·cm^–2^) couples. On the other hand, polymers **P18** and **P19** (Figure 14) are promising materials for the fabrication of phosphorescent organic light-emitting diodes (PHOLEDs) [49]. Both polymers can effectively absorb energy from the host properly, which is one of the prerequisites to realize high-performance OLED devices. Both polymers showed high performance (luminance = 2708 and 3356 c·dm^−2^, EQE = 0.5% and 0.67%, luminance efficiency = 0.6 and 0.55 L·mW^−1^ for **P18** and **P19**, respectively) with orange-yellow color emission at 10% doping level.

**Figure 15:**
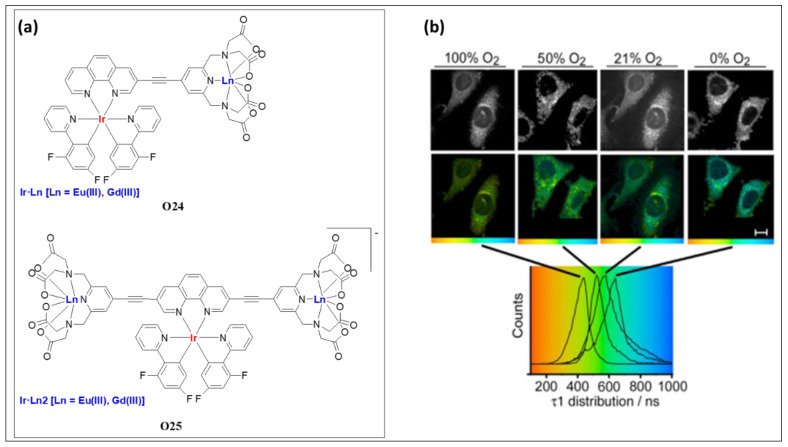
(**a**) Ir(III)/Ln(III) di- and trinuclear complexes (**O24** and **O25**). (**b**) Two-photon PLIM imaging of **O24** (Ln = Gd) stained Hela cells under different concentrations of oxygen. Reproduced with permission from ref. [76].

The application of luminescent lanthanide complexes for dual imaging (magnetic resonance imaging and optical imaging) is well established [79]. Jana and co-workers [76] showed that merging the high relaxivity feature of lanthanide ions with the luminescence from the Ir(III)/Ln(III) centers is an intriguing way to produce highly efficient bioimaging probes. The reported heterometallic complexes with *d*→*f* energy transfer feature exhibit high aqueous solubility, high intake in cancerous cell lines, low toxicity, and lysosomal target ability. Moreover, it was also reported that dinuclear complexes (**O24**, Figure 15a) were better than their trinuclear counterparts (**O25**, Figure 15a). One of the reported dinuclear complexes (**O24**: Ln = Gd) showed multi-modal imaging capability, which is the first of its kind. The luminescence lifetime of this multi-modal imaging probe varies inversely with the concentration of O_2_ (Figure 15b).

**Figure 16:**
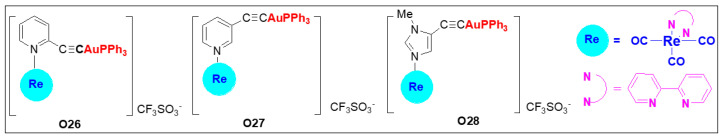
Heterometallic Re(I)/Au(I) complexes (**O26**–**O28**) with potential for cell imaging and cancer therapy.

On the contrary, nucleus and nucleolus localizing ability was reported for heterometallic Re(I)/Au(I) complexes (**O26**–**O28**, Figure 16) [77]. Compared to monometallic complexes (IC_50_ = 120–200 μm), higher cytotoxicity was exerted by the heterometallic derivatives (IC_50_ = 4.4–19 μm).

**Figure 17:**
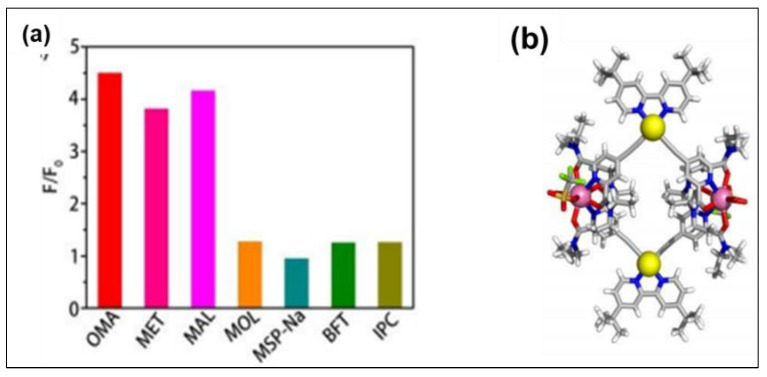
(**a**) Luminescence response to various pesticides, and (**b**) X-ray crystal structure of **O29** (C, gray; H, white; N, blue; O, red; S, orange; F, light green; Pt, yellow; Eu, pink). Reproduced with permission from ref. [80].

Researchers also explored the pesticides/chirality sensing ability of heterometallic complexes. Zhu and coworkers [80] exploited the luminescent enhancement by post-assembly modification of complex **O29** (Figure 17) for the sensing of some common pesticides. They reported that self-assembled coordination-driven complexes show a high propensity for thiophosphonates (OMA, MET and MAL) and turn-on the luminescence via displacement mechanism leading to intensity enhancement (~ 4-fold). However, negligible responses were noted for other pesticides. Overall, heterometallic complexes with open metal sites offer a new design principle in constructing sensors. Very recently, heterometal-organic macrocycles were also reported as having the ability to detect enantiomeric excess (ee) [81].

## 4. Conclusions

In summary, we reviewed the structure, properties and applications of metalla-ynes and poly(metalla-ynes) containing one or more types of metal ions. Both *d*-*d* and *d*-*f* combinations (either in the main- or side-chain) were briefly summarized. To date, a number of luminescent materials that contain *d*-*d* and *d*-*f* combinations (either in the main- or side-chain) separated by mono-, oligo- and poly-ynes have been developed and studied. From the discussion, it is quite clear that the combination of two metal centers, especially transition and lanthanide metals, is an effective way to modulate the PL properties. In the majority of cases, transfer of energy takes place from transition metal to lanthanide core, hence enhancing the luminescence intensity. Often, the hybrid material exhibits luminescence properties in between the transition and the lanthanide metal. In addition, owing to the wide variety of auxiliary groups, solution-processable small and large macromolecules can be realized with applications ranging from OLEDs through sensing to imaging. Despite multiple advantages, challenges such as limited synthesis incorporating metals other than the discussed ones, a mixture of products, batch-to-batch variation in the performance especially for polymeric materials are some of the main obstacles in this field.

## Figures and Tables

**Table 1:** Photoluminescence (PL) data of some selected heterometallic poly(metalla-ynes) **P1–P3** and **P6–P11**.

Code	Metals	Molecular Weight (×10^4^)		*λ_abs_* (nm)	*λ_ems_* (nm)	Lifetime of S_1_ (τ^a^, ns)	Lifetime of T_1_ (τ^b^, µs)	Φ (%)	E_g_ (eV)	Ref.
		*M*_w_	*M*_n_							
**P1**	Pt/Hg	2.9	1.5	386	409 ^a^, 542 ^b^,582 ^b^	1.23	194.38, 202.84	0.52	3.01	[46]
**P2**	Pd/Hg	0.45	0.40	411	430 ^a^, 544 ^b^, 578 ^b^	0.92	12.19, 22.71	12.60	2.85	[47]
**P3**	Pt/Pd	2.5	1.3	415	437 ^a^, 540 ^b^, 588 ^b^	0.89	193.7, 188.2	0.60	2.83	[47]
**P6**	Pt/Au	-	2.4	270, 276, 305, 319, 345, 362, 387	417 ^a^, 438^a^, 450^a^, 539 ^a^, 548 ^b^, 587 ^b^, 625 ^b^, 642 ^b^	0.71	181.63	1.03	2.98	[22]
**P7**	Pt/Au	-	2.7	264, 276, 316sh, 377	504^a^, 504 ^b^, 545 ^b^, 563 ^b^	10370	130.80	0.62	3.00	[22]
**P8**	Pt/Au	-	2.9	253, 262, 276sh, 317, 335	405 ^a^, 425 ^a^, 455 ^a^, 495 ^a^, 534 ^a^, 456 ^b^, 492 ^b^, 507 ^b^, 527 ^b^	0.99	44.31	0.27	3.19	[22]
**P9**	Pt/Au	-	2.1	275, 305, 319, 344, 361, 384	412 ^a^, 432 ^a^, 449sh ^a^, 542 ^a^, 584 ^a^, 548 ^b^, 586 ^b^, 619 ^b^	0.63	137.84	1.66	3.00	[22]
**P10**	Pt/Au	-	2.4	268, 275, 314sh, 373	504 ^a^, 503 ^b^, 529 ^b^, 603 ^b^	6460	165.23	1.69	3.01	[22]
**P11**	Pt/Au	-	3.1	253, 261, 316, 334	402 ^a^, 421 ^a^, 438 ^a^, 455 ^a^, 503 ^a^, 457 ^b^, 493 ^b^, 507 ^b^	0.72	40.14	0.30	3.12	[22]

*M_w_*: average weight molecular weight, *M_n_*: number weight molecular weight, *λ_abs_*: absorption wavelength peaks, *λ_ems_*: emission wavelength peaks, sh: shoulder, a: measured at 298 K, b: measured at 77 K, Φ: quantum yield, E_g_: energy gap.

## Data Availability

Not applicable.

## Abbreviations

acac	Acetylacetone
Bipy	Bipyridine
D-A	Donor-acceptor
EET	Electronic energy transfer
EQE	External quantum efficiency
E_g_	Energy gap
GPC	Gel permission chromatography
HOMO	Highest occupied molecule orbital
ISC	Inter system crossing
Ln(III)	Trivalent lanthanide
LUMO	Lowest unoccupied molecular orbital
MLCT	Metal to ligand charge transfer
*M_n_*	Number average molecular weight
*M_w_*	Weight average molecular weight
NLO	Non-linear optical
NMR	Nuclear magnetic resonance
O-E	Optoelectronic
OLEDs	Organic light-emitting diodes
OPL	Optical power limiting
phen	Phenyl
PHOLEDs	Phosphorescent organic light-emitting diodes
PL	Photo-luminescence
PLQY	Photo-luminescence quantum yields
RT	Room-temperature
SOC	Spin-orbit coupling
THF	Tetrahydrofuran
UV	Ultraviolet
Vis	Visible
τ	lifetime
ϕ	Quantum yield
ε	Absorption coefficient
$\lambda_{max}^{em}$	Wavelength of maximum emission
$\lambda_{max}^{abs}$	Wavelength of maximum absorption
